# Congenital diverticulum of the vermiform appendix: a rare finding of a routine operation

**DOI:** 10.1093/jscr/rjad116

**Published:** 2023-03-14

**Authors:** Maria Padar, Vijitha P Mudalige

**Affiliations:** Surgical Department, Bundaberg Base Hospital, Bundaberg Central, QLD, Australia; Surgical Department, Bundaberg Base Hospital, Bundaberg Central, QLD, Australia

## Abstract

Congenital diverticulum of the appendix as well as diverticulitis of the vermiform appendix remain a rare, mainly retrospective diagnosis, reported by the pathologist, post-appendicectomy. In clinical practice, however, it is important to be aware of this entity, the different types, diagnosis, association with malignancy and treatment options. We are presenting a young female case of pathologically proved true diverticulum of the appendix with associated uncomplicated appendicitis and an overview of the literature.

## INTRODUCTION

Diverticulosis and diverticulitis of the appendix is not as widely known, taught and recognized as the same pathology of the colon and small intestine. This is a case study of a young female with congenital, true diverticulum of the appendix, who presented with typical symptoms of appendicitis. Intraoperative findings of diverticulum of the inflamed appendix prompted further perusal of the surgical literature.

## CASE PRESENTATION

An 18-year-old otherwise healthy female presented to the emergency department of a public hospital with a 2-day history of abdominal pain. Her pain was constant, initially around the umbilicus, which moved to the right iliac fossa. The patient had no changes in bowel habits and had no urinary symptoms. Abdominal and pelvic ultrasound was requested, which revealed a tubular blind ending structure in the right iliac fossa, measuring 8 mm with evidence of thickening of the wall and surrounding free fluid. Free fluid was also found in the pelvis and in the pouch of Douglas. Inflammatory markers, including C-reactive protein and white cell count, were normal with mild neutrophilia. No other abnormalities were found. The patient was subsequently referred for a surgical review and a decision was made to proceed with a laparoscopic appendicectomy.

**Figure 1:**
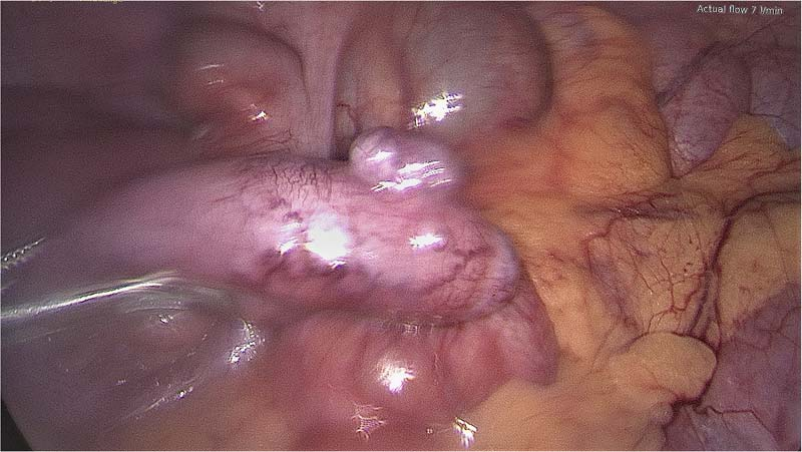
Appendix diverticulum, intraoperative photo.

**Figure 2:**
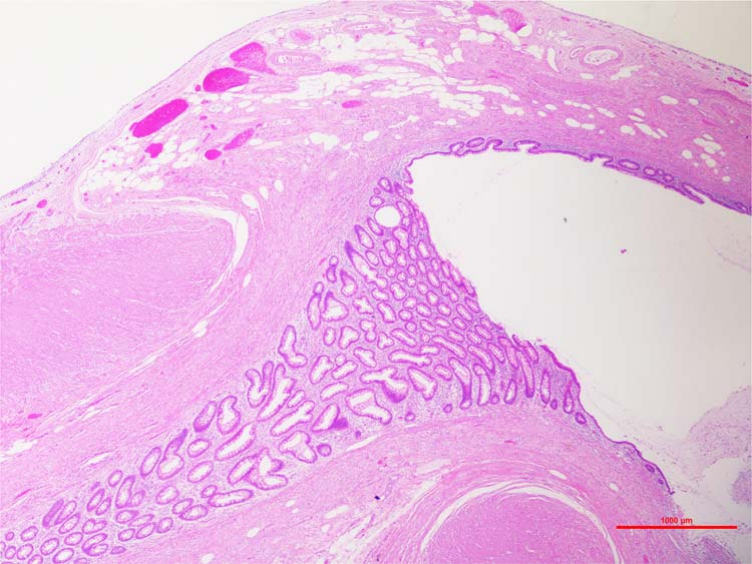
Hematoxylin and eosin staining, ×20, showing the tip of the appendix. Outpouching of the appendiceal mucosa through the muscular wall.

The procedure found an inflamed appendix with a 3-mm nodule on the tip of it (Fig. 1). No lymphadenopathy was seen. The uterus and ovaries were normal. The appendicectomy was carried out without complications and the patient was discharged the next day. Histopathology report showed an acute, suppurative appendicitis without malignant features. It also revealed a nodular lesion at the tip, representing a diverticular pouch, which further smooths muscle immune stain studies confirmed as a true diverticulum (Fig. 2).

## DISCUSSION

True diverticulum of an inflamed appendix is a very rare finding during routine appendicectomy of acute appendicitis in young adults. Diverticulitis of the appendix was first described by British physician Theophilus Nicholas Kelynack [1]. The incidence of diverticulosis of the appendix is 0.004–2% of surgical appendectomy specimens and 0.20 and 0.66% of autopsy studies [2]. After studying 50 000 autopsy and appendicectomy specimens, Collins revealed a collective incidence of 1.4%. True congenital diverticula, on the other hand, are even more sporadic of one case per 25 000 (0.004%) [2–4].

While acquired or pseudodiverticula are the most common diverticula of the appendix, true or congenital diverticula are a rare entity. Congenital or true diverticulum contains all three layers of the histologically normal wall with a very rare overall incidence of 0.014% and an average age of 31 years. They make up ~3% of all appendiceal diverticula. It usually presents as a single diverticulum on the antimesenteric border of the appendix. It has less complications, such as perforation, when inflamed compared with pseudodiverticula because of their protective, complete muscle coat [2].

Congenital diverticulum is recognized as a developmental abnormality. While its pathogenesis remains unclear, several mechanisms may be involved. Failure of recanalization of appendiceal lumen, duplication of the appendix, failure of obliteration of vitelline duct or adhesion between the appendix and the plica vascularis can be possible etiologies of congenital diverticulum [2]. There is also an association with trisomy D (13–15) or Patau syndrome [5, 6].

The features of diverticulum can only be recognized after pathological examination. Diagnosis of non-inflamed congenital appendix diverticulum is rare, as it does not cause symptoms, therefore it mainly remains as an incidental finding [2]. It is also found to have been markedly under reported, because of their small size and obliteration by inflammation at the time of resection [4].

Acquired or pseudodiverticula have a higher risk of perforation and association of malignancy, including neuroendocrine tumors, and mucinous adenocarcinoma [7, 8]. Moreover, appendiceal diverticulitis has a different clinical picture to appendicitis, such as intermittent, insidious pain in older patients, associated with a higher perforation rate [8]. Clinical differentiation is difficult, however, unless a CT, or in some rare cases an ultrasound, or an MRI would reveal a clear diagnosis, to be confirmed later by the pathology results of the appendicectomy specimen.

## CONCLUSION

Diverticulum of the appendix with or without inflammation is a rare entity. It is not always discovered before proceeding with surgical management of appendicitis. However, it is very important to recognize and to be aware of the different types, congenital or acquired, their pathology, symptomatology and their association with the well-documented complication of perforation and associated malignancies. It is also advisable to carefully check the final histopathology report.
